# Autosensitized Autogenous Vaccines

**Published:** 1915-12

**Authors:** 


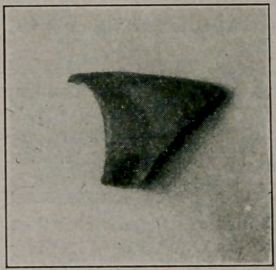

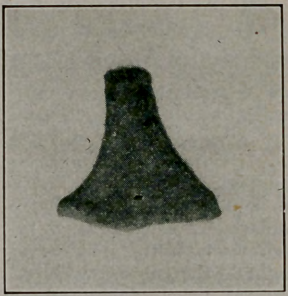

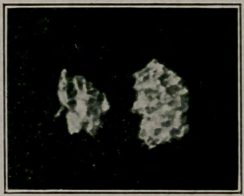

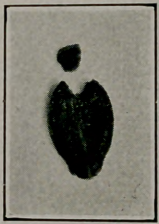

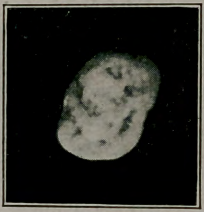

**Autosensitized Autogenous Vaccines.** In the October Clinics Dr. John B. Murphy again calls attention to the value of autosensitized vaccines. In order to obtain the best results from vaccines, not only should the micro-organisms come from the patient himself, but this autogenous strain should be cultivated upon the patient's own blood-serum, thus giving an automedium. The organisms may now often be recovered from the blood stream itself by the new Rosenau methods of citrating the blood and taking out the red blood corpuscles. If it is absolutely impossible to obtain the special strain of organism that is causing the trouble, then it is permissible to use a stock culture, but cultivated on the patients' own blood serum thus autosensitizing it. The dose can be made very much larger in these autosensitized vaccines.—C. W. H.

